# Intensity-Modulated Radiotherapy (IMRT) vs Helical Tomotherapy (HT) in Concurrent Chemoradiotherapy (CRT) for Patients with Anal Canal Carcinoma (ACC): an analysis of dose distribution and toxicities

**DOI:** 10.1186/s13014-015-0398-4

**Published:** 2015-04-17

**Authors:** Rosanna Yeung, Yarrow McConnell, Heather Warkentin, Darren Graham, Brad Warkentin, Kurian Joseph, Corinne M Doll

**Affiliations:** Department Oncology, Division of Radiation Oncology, University of Calgary, Tom Baker Cancer Center Calgary, 1331- 29th Street NW, Calgary, Alberta T2N4N2 Canada; Department of Surgery, University of Calgary, Foothills Medical Center, North Tower 10th Floor,1403- 29th Street NW, Calgary, Alberta T2N 2T9 Canada; Department of Oncology, Division of Medical Physics, Cross Cancer Institute, University of Alberta, 11560 University Ave NW, Edmonton, Alberta T6G 1Z2 Canada; Department of Oncology, Division of Radiation Oncology, Cross Cancer Institute, University of Alberta, 11560 University Ave NW, Edmonton, Alberta T6G 1Z2 Canada

**Keywords:** Anal cancer, Tomotherapy, Intensity modulated radiotherapy, Dosimetry, Toxicities

## Abstract

**Purpose:**

Intensity-modulated radiotherapy (IMRT) and helical tomotherapy (HT) have been adopted for radiotherapy treatment of anal canal carcinoma (ACC) due to better conformality, dose homogeneity and normal-tissue sparing compared to 3D-CRT. To date, only one published study compares dosimetric parameters of IMRT vs HT in ACC, but there are no published data comparing toxicities. Our objectives were to compare dosimetry and toxicities between these modalities.

**Methods and materials:**

This is a retrospective study of 35 ACC patients treated with radical chemoradiotherapy at two tertiary cancer institutions from 2008–2010. The use of IMRT vs HT was primarily based on center availability. The majority of patients received fluorouracil (5-FU) and 1–2 cycles of mitomycin C (MMC); 2 received 5-FU and cisplatin. Primary tumor and elective nodes were prescribed to ≥54Gy and ≥45Gy, respectively. Patients were grouped into two cohorts: IMRT vs HT. The primary endpoint was a dosimetric comparison between the cohorts; the secondary endpoint was comparison of toxicities.

**Results:**

18 patients were treated with IMRT and 17 with HT. Most IMRT patients received 5-FU and 1 MMC cycle, while most HT patients received 2 MMC cycles (p < 0.01), based on center policy. HT achieved more homogenous coverage of the primary tumor (HT homogeneity and uniformity index 0.14 and 1.02 vs 0.29 and 1.06 for IMRT, p = 0.01 and p < 0.01). Elective nodal coverage did not differ. IMRT achieved better bladder, femoral head and peritoneal space sparing (V30 and V40, p ≤ 0.01), and lower mean skin dose (p < 0.01). HT delivered lower bone marrow (V10, p < 0.01) and external genitalia dose (V20 and V30, p < 0.01). Grade 2+ hematological and non-hematological toxicities were similar. Febrile neutropenia and unscheduled treatment breaks did not differ (both p = 0.13), nor did 3-year overall and disease-free survival (p = 0.13, p = 0.68).

**Conclusions:**

Chemoradiotherapy treatment of ACC using IMRT vs HT results in differences in dose homogenity and normal-tissue sparing, but no significant differences in toxicities.

## Introduction

Since the 1980s, standard management of anal canal carcinoma (ACC) has been definitive chemoradiation therapy (CRT), with salvage abdominal-perineal resection (APR) for those who fail CRT [1-3]. Although CRT has been demonstrated in several randomized controlled trials as effective treatment for ACC, it is associated with significant toxicities [4-6]. Treatment breaks in up to 40-50% of patients have been reported due to hematological, dermatological and gastrointestinal toxicities [7,8]. Efforts have been made to reduce toxicities through newer chemotherapy regimens and radiotherapy (RT) techniques [9-11]. Development of more conformal RT techniques has reduced normal tissue toxicity and the need for unintended treatment breaks [11].

RTOG 0529 demonstrated significantly lower hematological, gastrointestinal and dermatological toxicities with intensity-modulated radiotherapy (IMRT) compared to conventional 2D-planning in ACC treatment [12]. Helical tomotherapy (HT) delivery, a newer RT technique, has shown improved target conformality, dose homogeneity and normal-tissue sparing compared to IMRT in other tumor sites [12,13].

In Alberta, use of IMRT vs HT is dependent on center availability and preference. To date, only one published study compares dosimetric parameters of IMRT vs HT in ACC, but there are no published data comparing toxicities [14]. Our objectives were to compare dosimetry and toxicities between these modalities.

## Materials and methods

### Patient population

This retrospective study included ACC patients treated with definitive CRT between 2008–2010 at two provincial tertiary cancer centers (Tom Baker Cancer Center (TBCC) and Cross Cancer Institute (CCI)). Approval for this study was obtained from the University of Calgary Conjoint Health Research Ethics board.

Patients were included if they were ≥18 years of age, had a histologic diagnosis of ACC, no other active malignancies, and were treated with curative-intent CRT with a primary planning target volume (PTV_primary_) dose of 54–55.4 Gy. All patients were treated with IMRT or HT, and chemotherapy consisting of 2 cycles of 5-FU and 1–2 cycles of mitomycin C (MMC) or cisplatin. Patients who had metastatic disease, received PTV_primary_ dose <54 or >55.4 Gy, RT alone, and RT treatment techniques other than IMRT or HT were excluded.

Pre-treatment evaluation of all patients included tumor biopsy, clinical examination, baseline complete blood count (CBC), and computed tomography (CT) abdomen and pelvis. Tumor size was based on clinical exam (when documented) or imaging. Weekly CBC and toxicities (skin, gastrointestinal, genitourinary) while on treatment were graded using the RTOG acute scoring index [15]. All blood counts were retrieved from the provincial clinical database (Alberta Netcare) during CRT and up to four weeks post last chemotherapy cycle. Hematological nadirs were recorded and analyzed.

### Volume definitions

Treatment plans were evaluated on an Eclipse workstation (Eclipse™ v8.9, Varian Medical Systems, Palo Alto, CA) without change to original planning target volumes delineated by treating physicians. Gross tumor volume (GTV), clinical target volume (CTV), planning target volume (PTV) were contoured, following the RTOG 0529 protocol, with deviations, if necessary, based on clinical judgment [11]. CTV_primary_ included the primary tumor and involved lymph nodes >1 cm identified on CT imaging and/or endoscopic ultrasound, plus a margin. CTV_nodes_ included regional lymph nodes at risk including peri-rectal, internal iliac, external iliac, obturator, presacral and inguinal lymph nodes. PTV_primary_ and PTV_node_ were generated with a uniform 1 cm margin around the CTV_primary_ and CTV_node_ respectively.

Organs at risk (OARs) included the bladder, peritoneal cavity, femoral heads, external genitalia (vulva in women, penis and scrotum in men), skin and bone marrow. These volumes were contoured on the existing plans. OAR volume definitions were based on the RTOG 0529 protocol except for small bowel and iliac crests [11]. The peritoneal cavity included large and small bowel from the L4/5 junction to the level of the bladder dome, with exclusion of named structures. In lieu of iliac crests, bone marrow was delineated, consisting of the L5 vertebra, sacrum, and bilateral iliac crests. This was felt to better reflect bone marrow dose from SPECT bone marrow imaging and IMRT bone marrow sparing studies [16,17]. Skin was generated as a 5 mm thick layer of tissue within the body contour, excluding PTV.

### Plan evaluation

The homogeneity of each plan with respect to PTV_primary_ and PTV_node_ was evaluated by the homogeneity (HI) and uniformity index (UI). HI was defined as the difference between D_max_ and D_min_ divided by the prescription dose, while UI was D_5_/D_95_. A value approaching 0 for HI and unity for UI indicates optimal dose homogeneity [18,19]. For OARs, median and mean dose were reported. In addition, V30, V40, and V50 were recorded for the femoral heads, bladder and peritoneal cavity. For external genitalia, V20, V30, and V40 were recorded while V10 and V20 were recorded for bone marrow.

### Statistical analysis

Patients were classified into two treatment cohorts: IMRT vs HT. A dosimetric comparison of plan homogeneity and OARs was performed. Disease-free survival (DFS), overall survival (OS), colostomy-free survival (CFS) and acute hematological and non-hematological toxicities were analyzed.

DFS was the interval between diagnosis and evidence of local, regional or metastatic failure, second primary, death or last follow-up for patients who did not fail. Local failure was evidence of persistent local disease or local recurrence. Regional failure was persistence, appearance or recurrence of regional nodal disease. Patients with persistent disease were considered as failing on the day of their first follow-up post CRT or date of biopsy-proven persistent disease (when available). Failure for OS was death due to any cause and was measured from diagnosis to the date of death or last follow-up. CFS was the interval between diagnosis and date of colostomy, including diverting colostomy and colostomy from salvage APR, or last follow-up for those not requiring a colostomy.

Results were analyzed with STATA Version 12.0 for Microsoft Windows (StataCorp LP, College Station, Texas). Chi-squared or Fisher exact testing was used to test differences between discontinuous variables. Wilcoxon rank-sum testing was used to test differences between continuous variables. DFS, OS, and CFS were analyzed using Kaplan-Meier survival analyses with log rank testing. *P* values of <0.05 were considered statistically significant.

## Results

### Demographics

72 patients were diagnosed with ACC in Alberta between 2008–2010. Thirty-seven patients were excluded from the analysis (13 treated with a technique other than IMRT or HT, 7 received RT alone, 1 was treated with surgery alone, 1 had metastatic disease at diagnosis, 13 received a dose PTV_primary_ <54 Gy or >55.4 Gy, and 2 with missing data). Of the remaining 35 patients, 18 patients were treated with IMRT (all treated at TBCC) and 17 with HT (all treated at CCI). Patients treated with HT were treated on TomoTherapy® Hi-Art® system, version 2.2.4.1 (Accuray, Inc, Sunnyvale, CA). Patients treated with IMRT were treated on, Clinac 21EX, Clinac IX, or Triology (Varian Medical, Palo Alto, CA). Patient, tumor and treatment characteristics are summarized in Table 1.

**Table 1:** **Baseline characteristics of ACC patients treated with chemoradiation by treatment cohort**

**Characteristic**	**IMRT (N = 18) n (%)**	**HT (N = 17) n (%)**	P-value*
Treatment center (TBCC/CCI)	18 (100%)/0 (0%)	0 (0%)/17 (100%)	<0.001
Age, y (median(range))	61 (45.1, 85.1)	52 (34.8, 69.7)	0.0045
Gender, (male/female)	6 (33.3%)/12 (66.7%)	4 (23.5%)/13 (76.5%)	0.71
Smoker	4 (22.2%)	11 (64.7%)	0.02
ECOG status			
ECOG 0	4 (22.2%)	7 (41.2%)	0.29
ECOG ≥1	14 (77.8%)	10 (58.8%)	
Histology			
Squamous	17 (94.4%)	17 (100%)	1.00
Other	1 (5.6%)	0 (0%)	
AJCC T-stage			
1	1 (5.6%)	0 (0%)	1.00
2	8 (44.4%)	7 (41.2%)	
3	7 (38.9%)	8 (47.1%)	
4	2 (11.1%)	2 (11.8%)	
AJCC N-stage			
N0	14 (77.8%)	11 (64.7%)	1.00
N1-3	4 (23.2%)	6 (36.3%)	
Pretreatment Blood Counts			
(median(range))			
Hb (g/dL)	124.5 (101, 156)	141 (101, 163)	0.10
WBC (x10^9^/L)	7.8 (5.1, 14.3)	8.6 (5.2, 15.5)	0.70
Neutrophil (x10^9^/L)	5.7 (2.8, 10.3)	6.3 (2.7, 11.3)	0.60
Platelet (x10^9^/L)	287.5 (146, 525)	287 (167, 368)	0.96
RT dose to Primary Tumor, Gy	54 (54, 55.4)	54 (54, 54)	0.04
(median(range))			
Chemotherapy			
Cisplatin + 5FU	2 (11.1%)	0 (0%)	<0.001
MMC 1 cycle + 5FU	16 (88.9%)	1 (5.9%)	
MMC 2 cycles + 5FU	0 (0%)	16 (94.1%)	

*Fisher exact testing used where any cell n < 5, otherwise Chi-square testing used.

Both groups were balanced in regards to performance status, histology, T stage, N stage, and pre-treatment hematological parameters. The IMRT group had slightly older patients (p = 0.0045) and fewer smokers (p = 0.02). The median RT dose was the same between the groups, but dose was more variable in the IMRT group. Chemotherapy regimen was significantly different, with 16 patients in the IMRT group receiving 1 MMC cycle with 5-FU and 16 patients in the HT group receiving 2 MMC cycles with 5-FU (p < 0.001).

### Dosimetric outcomes

**Figure 1:**
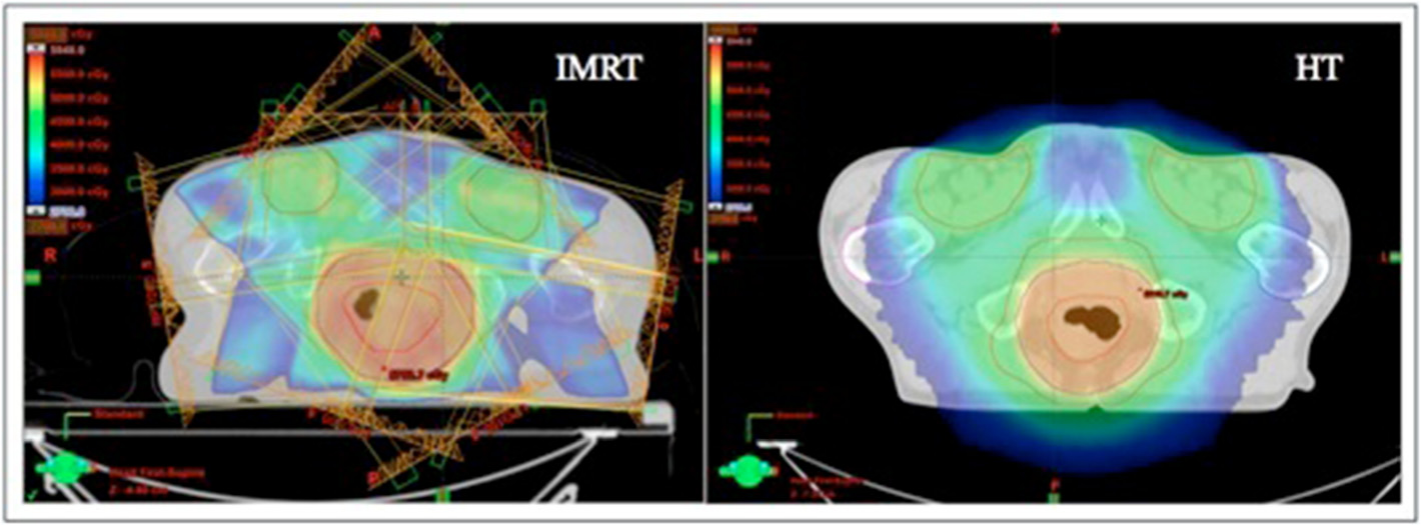
Dose distribution for ACC treated with IMRT vs HT.

Figure 1 shows a typical dose distribution on axial imaging at the level of PTV_primary_ for IMRT and HT techniques. Table 2 details the dosimetric coverage of treatment volumes and OARs by cohort. The HT group achieved more homogenous PTV_primary_ coverage compared to IMRT (HI p = 0.015, UI p < 0.001). PTV_nodes_ coverage did not differ between the techniques, although HI approached significance (p = 0.06). IMRT achieved better bladder, peritoneal space and femoral head sparing (V30 and V40, p ≤ 0.01). Median and mean skin dose was also lower with IMRT (p < 0.001). HT delivered lower dose to bone marrow (V10, p < 0.01) and external genitalia (V20 and V30, p < 0.001). A dosimetry model was constructed containing the factors of HI and UI for PTV_primary_ and HI for PTV_nodes_. No factor was significant for either outcome on multivariate analysis.

**Table 2:** **Dosimetric coverage of treatment volumes and OARs by treatment cohort**

**Parameter**	**IMRT**	**HT**	P-value*
	**Median (range), Mean**	**Median (range), Mean**	
PTV_primary_			
HI	0.28 (0.10, 1.09), 0.51	0.14 (0.05, 0.36), 0.18	0.015
UI	1.06 (1.04, 1.52), 1.09	1.03 (1.01, 1.07), 1.03	<0.001
PTV_node_			
HI	0.42 (0.14, 1.25), 0.48	0.55 (0.33, 0.79), 0.54	0.06
UI	1.12 (1.04, 1.52), 1.16	1.17 (1.09, 1.21), 1.16	0.15
Bladder			
V30	94 (51, 100), 88.8	100 (94, 100), 99	0.009
V40	56.5 (13, 92), 55.5	96 (44, 100), 90.9	<0.001
V50	14 (0.9, 54), 17.5	28 (0.2, 35), 21	0.37
Mean (Gy)	40.9 (32.4, 49.5), 41.3	47 (38.3, 49.2), 46.5	0.002
Median (Gy)	41.6 (30.3, 51.1), 41.5	47.1 (37.1, 49.0), 46.5	0.002
Bone Marrow			
V10	82.5 (47, 98), 78.6	67 (63, 75), 67.4	0.007
V20	62.5 (22, 88), 60.9	56 (51, 60), 56	0.08
Mean (Gy)	27.6 (12.4, 37.4), 27.2	26.2 (23.1, 28.5), 26.3	0.39
Median (Gy)	29.4 (9.1, 44.3), 28.9	28.3 (21.1, 33.4), 28.3	0.81
Femoral heads			
V30	68.5 (15, 99), 63.7	91 (76, 100), 90.3	<0.001
V40	14.5 (0, 50), 15.3	47 (27, 67), 50	<0.001
V50	0 (0, 14), 1.2	0 (0, 26), 3.1	0.94
Mean (Gy)	32 (23.5, 41.9), 31.5	38.9 (35.4, 44.1), 39.1	<0.001
Median (Gy)	32.7 (23.6, 40.2), 31.6	39.3 (35.2, 43.2), 39.8	<0.001
Peritoneal Cavity			
V30	26.5 (0.1, 66), 30.7	46 (6, 76), 44.6	0.01
V40	11 (0.9, 53), 17.2	34 (2, 66), 32.5	0.003
V45	4.5 (0, 44), 9.2	22 (0.5, 57), 23.4	0.002
V50	0.9 (0, 17), 1.7	2 (0, 27), 4.1	0.01
Mean (Gy)	20.2 (4.7, 36.4), 20.4	26.3 (9, 40.6), 25.6	0.04
Median (Gy)	19.7 (2.3, 41.8), 19.3	26 (4.1, 45.7), 25.4	0.09
External Genitalia			
V20	98.5 (62, 100), 91	73 (48, 98), 73.7	<0.001
V30	64 (19, 99), 65	25 (6, 58), 27.1	<0.001
V40	27 (0, 86), 25.7	5 (0, 48), 9.8	0.02
V50	2 (0, 44), 7.2	0.9 (0, 39), 4.4	0.11
Mean (Gy)	33.6 (21.8, 47.6), 33.6	25.1 (18, 38.9), 26	<0.001
Median (Gy)	33.3 (23.9, 48.9), 33.6	23.4 (18.9, 35.2), 24	<0.001
Skin			
Mean (Gy)	17 (1.4, 21.3), 16	23.1 (11.2, 26.8), 22	<0.001
Median (Gy)	15.9 (11.9, 19.2), 15.9	20.4 (6, 23.4), 19.1	<0.001

*Mann–Whitney test reported.

### Toxicities

Acute hematological and non-hematological toxicities are presented in Table 3. The most common toxicities were leukopenia and skin reaction. Grade 2+ hematological and non-hematological toxicities were similar between the groups. There were no significant differences in lower or upper gastrointestinal, genitourinary and skin toxicities. Additionally, febrile neutropenia and unscheduled treatment breaks did not differ between the two groups.

**Table 3:** **Grade 2+ toxicities in ACC patients treated with chemoradiation by treatment cohort**

**Toxicity**	**Total**	**IMRT**	**HT**	P-value*
	**(N = 35)**	**(N = 18)**	**(N = 17)**	
	**n (%)**	**n (%)**	**n (%)**	
Grade 2+ Hematologic Toxicities^†^				
Leukopenia	27 (77.1)	13 (72.2)	14 (82.4)	0.69
Neutropenia	22 (62.9)	10 (55.6)	12 (70.6)	0.36
Thrombocytopenia	13 (37.1)	6 (33.3)	7 (41.2)	0.63
Anemia	11 (31.4)	5 (27.8)	6 (35.3)	0.63
Febrile neutropenia requiring hospitalization	5 (14.3)	1 (5.6)	4 (23.5)	0.18
Grade 2+ Non-Hematologic Toxicities^†^				
Skin	29 (82.9)	14 (77.8)	15 (88.2)	0.66
Upper GI	9 (25.7)	6 (33.3)	3 (17.7)	0.44
Lower GI	18 (51.4)	12 (66.7)	6 (35.3)	0.06
GU	3 (8.6)	2 (11.1)	1 (5.9)	1.00
Unscheduled treatment break	8 (22.9)	6 (33.3)	2 (11.8)	0.23

*Fisher exact testing used where any cell n < 5, otherwise Chi-square testing used.

^†^RTOG acute radiation morbidity scoring criteria [17].

### Overall survival, disease-free survival and colostomy-free survival

**Figure 2:**
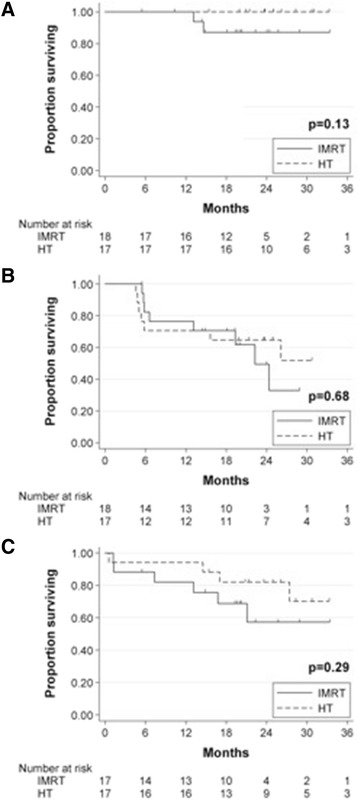
Survival analysis of ACC patients treated with chemoradiotherapy by treatment cohort. **A**. Overall Survival. **B**. Disease-Free Survival. **C**. Colostomy-Free Survival.

Median follow-up for survivors at the time of analysis was 23.7 months. Estimated 3-year OS (IMRT 87.1% vs HT 100%, p = 0.13, Figure 2A) and DFS (IMRT 32.9% vs HT 51.8%, p = 0.68, Figure 2B) by Kaplan Meier analysis were similar between the two groups. Estimated 3-year CFS was also similar (IMRT 57.3% vs HT 70.2%, p = 0.29, Figure 2C).

Multivariate analysis was not performed on OS, due to the small number of events. Multivariate analysis for DFS and CFS outcomes was limited to 3 factors per model, accounting for the number of events. For each outcome, a demographics model consisting of age, smoking status, and treatment group was constructed. No factor was significant for either outcome.

## Discussion

Advances in CRT for ACC have focused on toxicity reduction by alterations in chemotherapy regimens and radiotherapy delivery [9-11]. Large retrospective studies have shown promising results with cisplatin-based regimens compared to standard MMC treatment [20,21]. However, two recent phase III trials, RTOG 98–11 and ACTII, failed to demonstrate superiority of cisplatin to MMC for ACC treatment [9,10]. As such, ACC treatment with MMC and 5-FU remains standard of care.

Development of more conformal RT techniques has resulted in reduced normal-tissue toxicity. RTOG 0529 established superiority of IMRT to conventional 2D-planning in ACC treatment, with significant reduction in grade 2+ hematological, and grade 3+ gastrointestinal and dermatological toxicities using IMRT [11]. Newer RT techniques, such as HT, have shown improved target conformality, dose homogeneity and normal-tissue sparing compared to IMRT [12,13]. To date, there is only one study by Joseph et al. comparing dosimetry between HT and IMRT in ACC treatment [14]. Our current retrospective study serves as the first study comparing toxicities in ACC treatment between these two techniques.

Seventy-two patients were diagnosed with ACC between our study period, but less than half of the patients were included in the current analysis. The majority of exclusions were based on RT technique and PTV_primary_ dose outside of 54–55.4 Gy, in an effort to maintain consistency in dose and treatment volumes. Chemotherapy was significantly different, with the majority of IMRT patients receiving 1 MMC cycle, versus 2 cycles in the HT group. Historically, patients treated at the center where HT is available have been treated with 2 MMC cycles due to physician preference, versus 1 MMC cycle at the center where only IMRT is available.

Similar to the dosimetric study by Joseph et al., HT in our study achieved superior target conformality compared to IMRT for PTV_primary_ coverage [14]. OAR dose constraints in our study reflect those in RTOG 0529, with the exception of peritoneal cavity and bone marrow [11]. In both the current study and the previous study by Joseph et al., IMRT achieved better sparing of bilateral femoral heads, while HT delivered lower dose to external genitalia. However, in our study, IMRT also achieved lower bladder, peritoneal cavity and skin doses, while Joseph et al. reported improvement in sparing of bladder and peritoneal cavity with HT. In addition, our study demonstrated better bone marrow and external genitalia sparing with HT [14].

Differences in dose distribution and sparing of OARs between the two studies may be secondary to differences in calculation algorithms and study design. Although we found a significant difference between skin dose for IMRT and HT, it is known that there are large uncertainties in calculated skin dose for both the Eclipse analytic anisotropic algorithm and HT dose calculation algorithms [22,23]. Furthermore, the planning study by Joseph et al. generated HT and IMRT plans on the same patients, thereby minimizing anatomical variations between the two groups. There was also strict adherence to contouring and planning guidelines [14]. Our study, on the other hand, is retrospective with a larger sample of patients, and reflects real practice in a more generalized population.

Despite significant differences in OAR doses and chemotherapy regimens, acute hematological and non-hematological toxicities were similar between the groups. Three-year OS, DFS and CFS Kaplan Meier estimates were also similar. A recent retrospective study by our group suggests reduction of grade 3+ hematological and skin toxicities with 1 MMC cycle versus 2 cycles in ACC treatment, without compromise to OS, CFS and DFS [24]. Potential differences in acute toxicities secondary to different chemotherapy regimens may have been mitigated by radiotherapy technique. Further studies with more balanced groups in regards to chemotherapy may assist in confirming this hypothesis and our current results.

This study has inherent limitations of a retrospective study from a single health authority. Despite utilizing a standardized toxicity grading scale, potential subjectivity exists in retrospectively grading toxicities. In effort to obtain consistency, a single reviewer graded all toxicities.

## Conclusions

In our analysis of IMRT vs HT in ACC, differences in dose homogeneity and normal-tissue sparing were observed, but there were no significant differences in toxicities. Further investigation to increase cohort patient numbers should be performed, to definitively determine the impact of these techniques on toxicities and outcomes.

### Consent

Waiver of consent was granted by the local research ethics board, thus individual patient consents were not obtained. Patient identifiers were not used.
